# Variants of AbGRI3 carrying the *armA* gene in extensively antibiotic-resistant *Acinetobacter baumannii* from Singapore

**DOI:** 10.1093/jac/dkw542

**Published:** 2017-01-10

**Authors:** Grace A. Blackwell, Kathryn E. Holt, Stephen D. Bentley, Li Yang Hsu, Ruth M. Hall

**Affiliations:** 1 School of Life and Environmental Sciences, The University of Sydney, NSW 2006, Australia; 2 Centre for Systems Genomics, University of Melbourne, Parkville, Victoria 3010, Australia; 3 Department of Biochemistry and Molecular Biology, Bio21 Molecular Science and Biotechnology Institute, The University of Melbourne, Melbourne, Australia; 4 Wellcome Trust Sanger Institute, Hinxton, Cambridge, UK; 5 Saw Swee Hock School of Public Health, National University Health System, Singapore; 6 Yong Loo Lin School of Medicine, National University Health System, Singapore

## Abstract

**Objectives:** To investigate the context of the ribosomal RNA methyltransferase gene *armA* in carbapenem-resistant global clone 2 (GC2) *Acinetobacter baumannii* isolates from Singapore.

**Methods:** Antibiotic resistance was determined using disc diffusion; PCR was used to identify resistance genes. Whole genome sequences were determined and contigs were assembled and ordered using PCR. Resistance regions in unsequenced isolates were mapped.

**Results:** Fifteen GC2 *A. baumannii* isolated at Singapore General Hospital over the period 2004–11 and found to carry the *armA* gene were resistant to carbapenems, third-generation cephalosporins, fluoroquinolones and most aminoglycosides. In these isolates, the *armA* gene was located in a third chromosomal resistance island, previously designated AbGRI3. In four isolates, *armA* was in a 19 kb IS*26*-bounded transposon, designated Tn*6180*. In three of them, a 2.7 kb transposon carrying the *aphA1b* gene, designated Tn*6179*, was found adjacent to and sharing an IS*26* with Tn*6180.* However, in these four isolates a 3.1 kb segment of the adjacent chromosomal DNA has been inverted by an IS*26*-mediated event. The remaining 11 isolates all contained a derivative of Tn*6180* that had lost part of the central segment and only one retained Tn*6179*. The chromosomal inversion was present in four of these and in seven the deletion extended beyond the inversion into adjacent chromosomal DNA. AbGRI3 forms were found in available GC2 sequences carrying *armA.*

**Conclusions:** In GC2 *A. baumannii*, the *armA* gene is located in various forms of a third genomic resistance island named AbGRI3. An *aphA1b* transposon is variably present in AbGRI3.

## Introduction

Aminoglycoside antibiotics, particularly amikacin, gentamicin and tobramycin, are important treatment options when *Acinetobacter baumannii* infections are resistant to the first-line carbapenem antibiotics. Resistance to aminoglycosides can occur in a number of ways but most concerning is the emergence of acquired 16S rRNA methyltransferases that confer resistance to most aminoglycoside antibiotics. These enzymes provide ribosomal protection through methylation of 16S rRNA, which hampers the binding of aminoglycosides to the 30S subunit.^1^ ArmA is a 16S rRNA methyltransferase that was first characterized from a *Klebsiella pneumoniae* isolated in 2000 in France and shown to confer resistance to the 4,6-disubstituted deoxystreptamines, gentamicin, kanamycin, amikacin, tobramycin, isepamicin, netilmicin and sisomicin.^2^

The earliest *A. baumannii* isolates shown to carry the *armA* gene were recovered in 2003 in Korea.^3^ Since then, this gene has been reported in strains from North America,^4–6^ Japan,^7^ China,^8–10^ Malaysia,^11^ Nepal,^12^ India^13^ and Italy.^14^ It was also found in isolates from Norway recovered from patients that had previously been hospitalized in Asia.^15^ Where MLST data using the Institut Pasteur scheme are available, most are ST2 and are consequently members of global clone 2 (GC2), one of the globally disseminated clones of *A. baumannii*. In the earliest available completed genomes of GC2 isolates carrying *armA*, the gene is located in a 19 kb IS*26*-bounded transposon interrupting a chromosomal gene encoding a putative GNAT family acetyltransferase^5^^,^^6^ (corresponding to ABK1_1290 in 1656-2, GenBank accession number CP001921), designated *atr* hereafter.

Most resistant GC2 *A. baumannii* contain a form of both the AbGRI1 and AbGRI2 resistance islands. AbGRI1 characteristically contains the *sul2* (confers sulphonamide resistance), *strA*-*strB* (spectinomycin resistance) and *tetA*(B) (resistance to tetracycline) resistance genes.^16^^,^^17^ AbGRI2 variants on the other hand generally contain some or all of the following genes, *bla*_TEM_ (resistance to ampicillin), *aphA1b* in Tn*6020* (kanamycin and neomycin resistance)^18^ and *sul1*, *aacC1* and *aadA1* in a class 1 integron (conferring resistance to sulphonamides, gentamicin and streptomycin, respectively).^19–21^ Hence, the *armA* transposon represents a third chromosomally located resistance island, and for simplicity and consistency we recently called it AbGRI3.^20^ The three resistance islands are widely separated on the chromosome (see Figure 1 in Blackwell *et al.*^20^).

**Figure 1:**
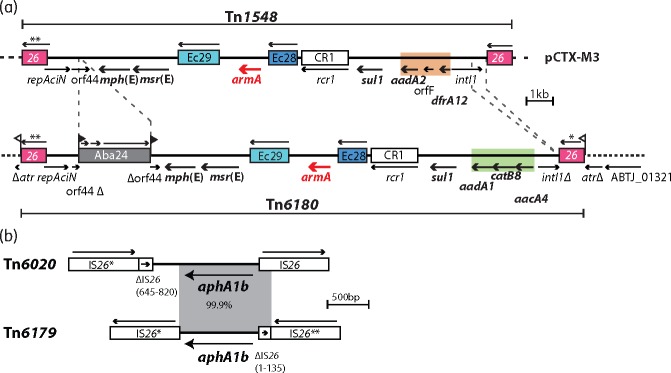
Transposon structures. (a) Comparison between Tn*1548* and Tn*6180*. (b) Comparison between Tn*6020* and Tn*6179*. Arrows below show the extent and orientation of the genes. Antibiotic resistance gene names are bold. IS and CR elements are depicted as coloured boxes and the numbers inside indicate the identity of the element. Arrows above the IS show the orientation of the *tnp* transposase gene. ISAba24 is 2421 bp in length with inverted repeats of 22 bp (21/22 bp) and creates an 8 bp TSD. An asterisk indicates the IS*26* is different to the standard sequence of IS*26* at three positions [G(459)A, G(613)A and G(614)A], and two asterisks indicates two differences [G(613)A and G(614)A]. Dashed lines indicate surrounding plasmid or chromosomal sequence. Thick black solid lines indicate sequence within the transposons. In (a), orange and green boxes highlight the different cassettes in each transposon. Flags indicate TSDs. Tn*1548* is drawn from pCTX-M3 (GenBank accession number AF550415). Tn*6180* is drawn to scale as in MDR-TJ (GenBank accession number CP003500) and the locus tag for the gene adjacent to *atr* is provided. In (b), grey blocks of shading represent sequence of 99.9% nucleotide identity. Tn*6020* is drawn to scale from GenBank accession number FJ172370 and Tn*6179* is drawn from GenBank accession number KX011025. This figure appears in colour in the online version of *JAC* and in black and white in the print version of *JAC*.

To date, the content and organization of the *armA* transposon in GC2 isolates has been described only as similar to that of Tn*1548*.^5^^,^^6^ However, there are several differences that distinguish it and we have named it Tn*6180* (Figure 1a). Compared with Tn*1548*, the Tn*6180* integron carries a different set of cassettes *aadA1*-*catB8-aacA4*, conferring resistance to streptomycin and spectinomycin, chloramphenicol and gentamicin and tobramycin, respectively, and a deletion at the right end of the transposon has removed 216 bp of the *intI1* gene. Another difference is the insertion of ISAba24, a novel member of the IS*66* family, in orf44 in Tn*6180*. In three completed GC2 genomes,^9^^,^^10^ Tn*6180* together with an *aphA1b*-containing segment has been reported as a plasmid. However, as the only putative *rep* gene present, *repAciN*, is incomplete and is known to be non-functional,^22^ this configuration is questionable.

In this study, 20 carbapenem-resistant GC2 isolates collected at Singapore General Hospital (SGH) between 1996 and 2011 were examined for the presence of *armA* and its context was analysed. Whether circular forms are present at detectable levels was also examined.

## Materials and methods

### Bacterial isolates

Twenty extensively antibiotic-resistant (XAR) *A. baumannii* isolates recovered from SGH and assigned to GC2 using tri-locus typing^23^ were examined. Fifteen of these, isolated over the period 2004–11, were found to contain the *armA* gene and were further characterized (Table 1). All isolates were screened for resistance to 30 antibiotics using a disc diffusion method as described previously.^21^ Colistin susceptibility was determined by Etest (bioMérieux, Durham, NC, USA).

**Table 1:** Aminoglycoside resistance of the GC2 isolates carrying *armA* from SGH used in this study

Isolate	Source^a^	Year	Amioglycoside resistance^b^								*aacA4*	*aphA1b*	AbGRI3 version
SGH0702	blood	2007	Ak	Gm	Km	Net	–	Sm	Sp	Tm	+	–	1i
SGH0402	ETTA	2004	Ak	Gm	Km	Net	Nm	Sm	Sp	Tm	+	+	2i
SGH0403	ETTA	2004	Ak	Gm	Km	Net	Nm	Sm	Sp	Tm	+	+	2i
**SGH0701**	blood	2007	Ak	Gm	Km	Net	Nm	Sm	Sp	Tm	+	+	2i
**SGH1111**	sputum	2011	Ak	Gm	Km	Net	–	Sm	Sp	Tm	+	–	3i
**SGH1112**	sputum	2011	Ak	Gm	Km	Net	–	Sm	Sp	Tm	+	–	3i
SGH0905	blood	2009	Ak	Gm	Km	Net	Nm	Sm	Sp	Tm	–	+	4
SGH0907	blood	2009	Ak	Gm	Km	Net	Nm	Sm	Sp	Tm	–	+	4
**SGH0908**	blood	2009	Ak	Gm	Km	Net	Nm	Sm	–	Tm	–	+	4
SGH1010	sputum	2010	Ak	Gm	Km	Net	–	Sm	Sp	Tm	–	–	4
SGH1011	blood	2010	Ak	Gm	Km	Net	–	Sm	Sp	Tm	–	–	4
SGH1012	ETTA	2010	Ak	Gm	Km	Net	–	Sm	Sp	Tm	–	–	4
SGH1113	blood	2011	Ak	Gm	Km	Net	Nm	Sm	Sp	Tm	–	+	4
SGH0825	trach	2008	Ak	Gm	Km	Net	Nm	Sm	Sp	Tm	+	+	5i
SGH0602	ETTA	2006	Ak	Gm	Km	Net	–	Sm	Sp	Tm	+	–	+^c^

a Tracheal aspiration (trach) and endotracheal aspiration (ETTA).

b Codes are as follows: amikacin, Ak; gentamicin, Gm; kanamycin, Km; netilmicin, Net; neomycin, Nm; streptomycin, Sm; spectinomycin, Sp; tobramycin, Tm.

c The junction on the left is the same as SGH0701 but then not positive for any other PCR.

### DNA isolation and PCR amplification

Genomic DNA for WGS was extracted using a Qiagen DNA extraction kit. Genomic DNA for PCR was prepared as described previously.^24^ For screening of the inverted arrangements, genomic DNA from single colonies was isolated by heating at 100ºC for 5 min as described previously.^25^ PCR was used to screen for resistance genes and to assemble the AbGRI3 region and the amplicons were sequenced. The primers used to detect the *aphA1b* and *aphA6*,^24^ ISAba1 upstream of *ampC*^26^ and *oxa23*^27^ genes have been reported previously. RH2012 (5'-TCCATTCCCTTCTCCTTTCC-3') and RH2013 (5'-GGGGGTCTTACTATTCTGCCTA-3') were designed to detect *armA*. The primers used to assemble AbGRI3 are listed in Table 2. All primers can be used with an annealing temperature of 60°C. The PCR conditions used to detect short amplicons (<2.5 kb) were as described previously using *Taq* DNA polymerase and a minimum extension time of 1 min per 1 kb of predicted amplicon length.^24^

**Table 2:** Primers used to assemble AbGRI3 variants

Primer^a^	Primer location^b^	Sequence (5'-3')
RH2001	ABK1_1291	GGAGTTGGTTTTGGTACAGCA
RH2002	rep*AciN*	TATAAGCCACCTCGCTCACC
RH2003	*intI1*	GCCTTGATGTTACCCGAGAG
RH2005	ABK1_1288	CACTGATCTGCTGGCTTTCA
RH2006	ABK1_1287	TGACGAGCTTTGTTTAGGTGTG
RH2010	ISAba24	TTTCGTGACACTCTCGCTTG
RH2011	*aacA4*	CATAGAGCATCGCAAGGTCA
RH2015	ABK1_1289	AATGTGGTTGGCGGTTTTTA
RH2016	ABK1_1289	GCAGCCTCAAAGTGGAAAAC
RH880	*aphA1b*	CAACGGGAAACGTCTTGCTC
RH831	*aphA1b*	TATACCCATATAAATCAGCATCC

a All but RH880^24^ were designed for this study.

b Gene or 1656-2 locus tags (GenBank accession number CP001921).

### DNA sequencing and sequence analysis

Genomic DNA of four isolates was sequenced using Illumina HiSeq at the Wellcome Trust Sanger Institute (associated data shown in Table S1, available as Supplementary data at *JAC* Online). Paired-end reads of 100 bp were assembled as described previously.^28^ Contigs of the draft genomes containing antibiotic resistance genes were identified using ResFinder (https://cge.cbs.dtu.dk//services/ResFinder/) and contigs containing fragments of IS*26* were recovered using standalone BLAST.^29^ Sequencher 5.2.3 (Gene Codes Corporation, Ann Arbor, MI, USA) was used to assemble the resistance regions and gene construction kit version 4 (Textco, West Lebanon, NH, USA) was used to draw figures to scale. In addition, MLST using the Pasteur and Oxford schemes (http://pubmlst.org/abaumannii/) was performed for the isolates with genome sequence available.

### GenBank accession numbers

The sequences of AbGRI3 variants and surrounding chromosomal sequence from SGH0701, SGH0908 and SGH1111 have been deposited into GenBank under accession numbers KX011025, KX011026 and KX011027, respectively. The reads and draft genomes have been deposited in the European Nucleotide Archive under Project Number ERP001080 (see Table S1 for individual accession numbers).

## Results

### Resistance phenotype and genotype

Twenty GC2 isolates from SGH were screened for the presence of a number of resistance determinants. All the isolates contained *oxa23*, conferring resistance to carbapenems and ISAba1 was found upstream of *ampC* conferring resistance to third-generation cephalosporins. The *armA* gene was found in 15 isolates, indicating that AbGRI3 may be present. The presence of the *aphA1b* (confers resistance to kanamycin and neomycin) and *aacA4* (amikacin, kanamycin and neomycin resistance) genes was variable (Table 1).

The 15 *A. baumannii* isolates carrying *armA* (Table 1) were all found to be XAR, using published criteria.^30^ They were resistant to carbapenems (imipenem, meropenem and doripenem), extended-spectrum cephalosporins (cefotaxime, ceftriaxone, ceftazidime and cefepime), penicillins and β-lactamase inhibitors (ticarcillin/clavulanic acid, ampicillin/sulbactam and piperacillin/tazobactam), quinolones (nalidixic acid), fluoroquinolones (ciprofloxacin and levofloxacin), folate pathway inhibitors (trimethoprim and sulfamethoxazole) and tetracyclines (tetracycline and doxycycline). All isolates were resistant to streptomycin, gentamicin, kanamycin, netilmicin, tobramycin and amikacin but varied in their susceptibility to neomycin and one isolate was susceptible to spectinomycin (Table 1). Resistance to neomycin correlated with the presence of *aphA1b*. SGH1010 had reduced susceptibility to colistin (MIC 3 mg/L) but the other isolates were all susceptible.

### Features of the sequenced isolates

The genomes of four isolates carrying *armA* were sequenced (bold in Table 1), and for these isolates, MLST was performed using both the Institut Pasteur and Oxford schemes (Table 3). Three isolates were ST2 (*cpn60-2*, *fusA-2*, *gltA-2*, *pyrG-2*, *recA-2*, *rplB-2*, *rpoB-2*) using the Institut Pasteur scheme and SGH0908 was a single locus variant (SLV) of ST2 (*cpn60-1*). Hence, all isolates belong to GC2, confirming the original screening. Using the Oxford scheme, two were ST208 (*cpn60-2*, *gdhB-3*, *gltA-1*, *gpi-97*, *gyrB-3*, *recA-2*, *rpoD-3*). SGH0701 was ST218, an SLV of ST208 (*gpi-102*). SGH0908 had a novel ST that was assigned the number ST1166. ST1166 is a triple locus variant (*cpn60-4*, *gpi-106* and *rpoD-2*). The *gpi* gene lies within the K locus^31^ and the differences in the *gpi* allele reflect the fact that the isolates carry three different sets of capsule biosynthesis genes (Table 3).

**Table 3:** Properties of sequenced GC2 isolates from SGH

Isolate	MLST (Pasteur)	MLST (Oxford)	*gpi* allele	K locus^a^	Resistance regions		
					location^b^	resistance genes	no. of contigs^c^
SGH0701	2	218^d^	102	7	AbGRI1	*sul2*, *strA-strB*, *tetA*(B)	2
					AbGRI2	none	
					**AbGRI3**	***armA***, *msr*(E), *mph*(E), *catB8*, *aadA1*, *aacA4*, *sul1*, *aphA1b*	2
					other	*oxa23*	1
SGH0908^e^	98^f^	1166^g^	106	49	AbGRI1	*sul2*, *strA-strB*, *tetA*(B)	2
					AbGRI2	*aphA1b*, *bla*_TEM_	2
					**AbGRI3**	***armA***, *msr*(E), *mph*(E)	1
					other	*oxa23*	1
SGH1111	2	208	97	2	AbGRI1	*strA-strB*, *tetA*(B)	1
					AbGRI2	*aacC1*	1
					**AbGRI3**	***armA***, *msr*(E), *mph*(E), *catB8*, *sul1*, *aacA4*, *aadA1*	1
					other	*oxa23*	1
SGH1112	2	208	97	2	AbGRI1	*strA-strB*, *tetA*(B)	1
					AbGRI2	*aacC1*	1
					**AbGRI3**	***armA***, *msr*(E), *mph*(E), *catB8*, *sul1*, *aacA4*, *aadA1*	1
					other	*oxa23*	1

a The structures of KL2, KL7 and KL49 are in GenBank accession numbers JN968483, KX011025 and KT359616, respectively.

b Other: genes that have been detected in plasmids and/or at various locations in the chromosome. SGH0701 and SGH0908 contain two and three copies of the *oxa23* gene, respectively, with one copy in AbGRI1.

c Contigs containing resistance genes only.

d SLV of ST208.

e Spectinomycin susceptible as no *aadA* gene is present.

f SLV of ST2, has *cpn60-1*.

g Trilocus variant of ST208, has *cpn60-4*, *rpoD-2*.

**Figure 2:**
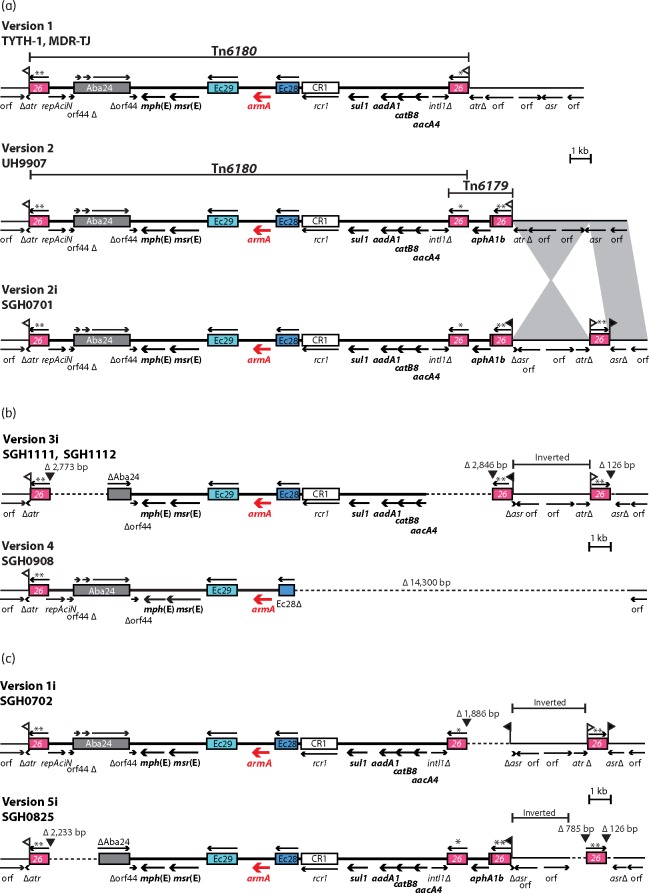
AbGRI3 structures. (a) Comparison of version 2i with published versions of AbGRI3. (b) AbGRI3 variant forms in the sequenced isolates from Singapore. (c) Variants characterized by mapping in the unsequenced isolates from Singapore. Thick lines represent sequence internal to the resistance island while thin lines indicate chromosomal sequence. Arrows below show the extent and orientation of the genes and antibiotic resistance genes are in bold. IS and CR elements are depicted as boxes with their numbers inside. Arrows above the IS show the orientation of the *tnp* transposase gene. An asterisk indicates the IS*26* is different to the standard sequence of IS*26* at three positions [G(459)A, G(613)A and G(614)A], and two asterisks indicates two differences [G(613)A and G(614)A]. Flags indicate the 8 bp TSDs. In (a), grey shading highlights regions of chromosomal sequence with >99.9% nucleotide identity. Version 2 was drawn to scale from GenBank accession number AY0H00000000. In (b) and (c), dashed lines show sequence that is missing and the size of the deletion is indicated along with a black triangle showing the predicted origin of the deletion. The region of inverted sequence is indicated above the line. This figure appears in colour in the online version of *JAC* and in black and white in the print version of *JAC*.

For each sequenced isolate, the resistance genes identified by ResFinder, and listed in Table 3, have been separated into four different lines based on where they are found, in AbGRI1, AbGRI2, AbGRI3 or elsewhere. Each of these isolates contains a version of AbGRI1 and AbGRI2 but SGH0701 has no resistance genes in AbGRI2. These resistance islands will be described in more detail elsewhere. The *aphA1b* gene in Tn*6020* is frequently found in AbGRI2.^18–20^ However, in the sequenced *aphA1b*-positive strains, the context of this gene is different (see Figure 1b). We named this 2706 bp transposon Tn*6179*. We found Tn*6179* in the genome of UH9907 (GenBank accession number AY0H00000000),^5^ adjacent to Tn*6180* and sharing one IS*26* with the right IS of Tn*6180*. This AbGRI3 structure, version 2, is shown in the second line of Figure 2(a). Hence, Tn*6179* was assigned to AbGRI3 in the SGH isolates sequenced here.

### AbGRI3 in SGH0701

SGH0701 contained a 17.5 kb and a 1.1 kb contig, matching the internal sequence of Tn*6180* and Tn*6179*, respectively, and flanked by fragments of IS*26*. Chromosomal contigs 133 and 3.1 kb in size, each contained part of *atr* directly adjacent to a fragment of IS*26* and the boundaries between IS*26* and the chromosome-derived sequence were the same as in AbGRI3 version 1 (found in MDR-TJ and TYTH-1) and version 2 (found in UH9907) (Figure 2a). The *repAciN* end of Tn*6180* was linked to the *atr* end of the 133 kb contig and the other end was connected to Tn*6179*. While it was possible to link the other side of Tn*6179* to the remainder of *atr* in the 3.1 kb contig, it is not clear if this is the actual arrangement or if the 3.1 kb segment is inverted as shown in Figure 2(a) (see below).

The 3.1 kb chromosomal contig, which matches *atr* at one end, is bounded by IS*26* at both ends. The other end matches a part of an aspartate racemase gene (corresponding to ABK1_1287 in 1656-2, GenBank accession number CP001921) or *asr* for simplicity. The remainder of *asr* was found adjacent to an IS*26* outer end on another 142 kb chromosomal contig. Therefore, an additional copy of IS*26* interrupts the *asr* gene in SGH0701. It appears that an inversion of 3.1 kb of the chromosome has been caused by the replicative intramolecular transposition^20^ of the IS*26* at the right end of AbGRI3 to a site in *asr*, creating an 8 bp target site duplication (TSD) and duplicating the IS*26* involved, and this configuration is shown as version 2i, where ‘i’ indicates the adjacent inversion in Figure 2(a). The sequence of the instigating IS*26* and the duplicated copy both differ from the standard sequence of IS*26* by the same two base pairs (IS26** in Figures 1 and 2), which is consistent with this mechanism (Figure 2a).

However, we were unable to assign unambiguously the orientation of the 3.1 kb segment. Using PCR, Tn*6179* could be linked to both ends of the 3.1 kb contig (primer RH831 with RH2005 or RH2015), and both ends of the 3.1 kb contig could also be joined to the *asr* end of the 142 kb contig (primer RH2006 with RH2005 or RH2015). These two orientations were confirmed by sequencing the amplicons. As the IS*26* at the ends of the 3.1 kb contig are in opposite orientation, the 3.1 kb segment could be flipping due to either homologous recombination or the action of IS*26*. To attempt to determine the major direction of the 3.1 kb segment, boiled DNA preparations for 10 single colonies of SGH0701 were checked for amplification of both orientations using long extension times that should allow polymerization to proceed through IS*26* and to prevent PCR artefacts (see the Materials and methods section). Though the re-inverted orientation could still be detected in each single colony, the inverted orientation yielded brighter products.

### AbGRI3 in SGH1111 and SGH1112

SGH1111 and SGH1112, both ST208 (Oxford) and both from 2011, had the same AbGRI3 structure, named version 3i. The IS*26* at the left end of Tn*6180* has caused a deletion that extends into ISAba24 (776/2421 bp remaining) (Figure 2b). The IS*26* on the right of Tn*6179* has removed *aphA1b*, the remainder of the *intI1* gene, the *attI1* site and 7 bp of the *aacA4* gene cassette, leaving the *aacA4* reading frame intact. Like version 2i, version 3i has an additional copy of IS*26* downstream of the resistance island and the 3.1 kb chromosomal sequence in-between can be detected in both orientations. However, part of *asr* is missing as another deletion, likely caused by the adjacent IS*26*, has removed 126 bp of this gene (Figure 2b).

### AbGRI3 in SGH0908

Version 4 of AbGRI3 in SGH0908 contains the standard boundary on the left but an unusual recombination event has caused the loss of 14 300 bp relative to version 2i (11 586 bp relative to version 1) (Figure 2b). The deletion extends from 696 bp into ISEc28 (201 bp lost) to chromosomal sequence 3112 bp to the right of the resistance island (Figure 2b) removing the invertible segment. There is no clear evidence to explain how this event occurred but an alignment revealed that there was a 4 of 5 bp identity [5'-AA(T)AGT-3'] between the end of the ISEc28 fragment and the chromosomal sequence that could have facilitated the recombination.

### Predicted structures of AbGRI3 in the unsequenced SGH isolates

Mapping strategies used to assemble AbGRI3 in the sequenced isolates were applied to predict the structures of AbGRI3 in the remaining *armA*-positive isolates (Table 1). SGH0402 and SGH0403 from 2004 also contained AbGRI3 version 2i. A further six isolates had AbGRI3 version 4. Two additional AbGRI3 variants were found, each in a single isolate (Figure 2c). SGH0702 contains version 1i, which like version 1 has only Tn*6180* (Figure 2). The simplest explanation for this configuration is that the IS*26* shared by Tn*6180* and Tn*6179* caused a deletion that removes the Tn*6179* translocatable unit (TU), which is a circular fragment of DNA with a single copy of IS*26*, as has been described elsewhere.^32^^,^^33^ Version 5i, found in SGH0825, had a smaller deletion at the left end of Tn*6180* relative to version 4, retaining 1316 of the 2421 bp ISAba24 (Figure 2b and c). Tn*6179* was also present but deletions in the chromosomal DNA on each side of the additional IS*26* were detected. The left side has lost 785 bp while the right has lost 126 bp and this deletion is also in version 3i. In both variant 1i and 5i, where the additional IS*26* interrupting the *asr* gene is present, the chromosomal segment in-between could be detected in both orientations. The complete structure of AbGRI3 in SGH0602 could not be determined using the PCR mapping strategy. The left junction of Tn*6180* is intact, and the sequence up to *armA* is present but no amplicon was produced for the remainder of the mapping PCRs.

### AbGRI3 in publicly available sequences

The finished genomes of MDR-TJ and TYTH-1 were the first found to contain a form of AbGRI3, which was comprised of just Tn*6180* (AbGRI3-1). Since then, the content of the AbGRI3 islands in NCGM_237^7^ and YU-612^34^ has been described. Other completed genomes were analysed in this study. Most included both Tn*6180* and Tn*6179* (AbGRI3-2), though others had only Tn*6180* or a deletion derivative of this transposon (Table 4). An IS*26* interrupting *asr* was not present in any of the sequences. Interestingly, PKAB07 (GenBank accession number CP006963) has the same deletion present as AbGRI3-4 and this version of AbGRI3 is also in isolate A072 (GenBank accession number KT354507).^35^

**Table 4:** Structures of AbGRI3 present in finished GC2 genomes

Isolate^a^	Year of isolation	GenBank accession number	Chromosome on the left	AbGRI3	Chromosome on the right
MDR-ZJ06	2006	CP001937	+	IS*26*^b^	– (Δ28147 bp)
MDR-TJ	<2011	CP003500	+	Tn*6180*	+
TYTH-1	2008	CP003856	+	Tn*6180*	+
BJAB07104	2007	CP003846	+	IS*26*^b^	+
BJAB0868	2008	CP003849	+	IS*26*^b^	+
XH860	2009	CP014538	+	IS*26*	– (Δ3338 bp)
XH856	2010	CP014541	+	Tn*6180*, Tn*6179*	+
ORAB01	2012	CP015483	+	Tn*6180*, Tn*6179*	+
KPN10P02143	2012	CP013924	– (Δ 2850 bp)	Tn*6180*, Tn*6179*	+
NCGM_237	2012	AP013357	– (Δ2814 bp)	Tn*6180*	– (Δ40215 bp)
PKAB07	2014	CP006963	+	ΔTn*6180*^c^	– (3937 bp)
XH386	2014	CP010779	+	Tn*6180*, Tn*6179*	+
YU-R612	2014	CP014215	– (Δ2850 bp)	two copies of ΔTn*6180*^d^	+

a The finished genomes with clearly discernible AbGRI3 regions have been included.

b These genomes include a Tn*6180*-Tn*6179* TU form said to be a plasmid.

c Two copies of IS*26* bound the left end of Tn*6180* and a deletion identical to version 4 of AbGRI3 is present.

d First copy of ΔTn*6180* contains the first 308 bp of *mph*(E) to 26 bp of *intI1* and the second copy of ΔTn*6180* contains same bases of *mph*(E) but instead has 364 bp of *intI1*.

A circular form of AbGRI3-2 (Tn*6180* and Tn*6179*) has been reported previously as a plasmid in three genomes, MDR-ZJ06,^10^ BJAB0868 and BJAB07104,^9^ though pMDR-ZJ06 carries the cassette array (*aacC1*-orfP-orfQ-*aadA1*) normally seen in the integron of AbGRI2. In the chromosome of BJAB0868 and BJAB07104, an IS*26* is present in the location of AbGRI3, i.e. interrupting the *atr* gene, and it is flanked by the same 8 bp TSD. MDR-ZJ06 also has a suitably located IS*26* but 22.8 kb of adjacent chromosomal sequence is missing. As it has been shown that *repAciN* is not a functional replication initiation protein,^22^ it seems unlikely that this form could exist stably as a plasmid. Hence, it is likely that the proposed plasmid forms instead belong in the chromosome, as AbGRI3.

### Can TUs be detected?

Recently, the AbGRI3-2 structure that includes both Tn*6180* and Tn*6179* was found in the isolate A071 (GenBank accession number KT317075) and named Tn*6279*.^35^ This study also reported that circular TU forms of AbGRI3-2 (Tn*6180* and Tn*6179* with only two copies of IS*26*) as well as just AbGRI3-1 (Tn*6180* with one copy of IS*26*) could be detected by PCR in this isolate. However, using DNA from SGH0701, which carries AbGRI3-2, and PCR conditions that allow polymerization through IS*26*, thereby avoiding PCR artefacts, no amplicons were detected for the circular forms of AbGRI3-2 (RH2002-RH831) or AbGRI3-1 (RH2002-RH2003). Hence, the possibility that the earlier observations were due to artefacts caused by short PCR extension times or other factors in the case of A071 should be investigated.

## Discussion

In several sequenced GC2 genomes, the *armA* gene is found in AbGRI3, the third resistance island typically found in this group. We have shown previously that ancestral forms of AbGRI1 and AbGRI2 were acquired by a GC2 isolate prior to 1982.^20^ The AbGRI3 island appears to have been acquired in the early 2000s by a GC2 isolate already carrying these two resistance islands, probably in Asia. The earliest isolate with the *armA* gene recovered at SGH was from 2004 and it contained AbGRI3-2. However, in most of the SGH isolates, a 3.1 kb segment of chromosome adjacent to the Tn*6179* end has been inverted and an IS*26* has been duplicated in the process. This arrangement is unique to the isolates from SGH and could prove useful in tracking their spread. A surprising finding was that the 3.1 kb segment could be detected in both orientations using PCR, and further experiments such as cloning the fragment or PacBio sequencing will be required to determine if the segment is actually inverting frequently.

The presence of an 8 bp TSD surrounding AbGRI3 demonstrates that, like AbGRI2,^20^^,^^36^ this island entered the chromosome of a GC2 isolate through the action of IS*26*. What is less clear is if both Tn*6180* and Tn*6179* entered together or individually.^33^ Based on the sequences of the IS*26* at the outer boundaries of AbGRI3-2, a likely option is that a TU derived from both Tn*6180* and Tn*6179* inserted into the *atr* gene by a Tnp26-mediated replicative transposition event, duplicating an IS26** and producing the TSD.

The AbGRI3 region has continued to evolve *in situ*. As for AbGRI2,^20^^,^^21^ this variation has mainly occurred through deletions adjacent to copies of IS*26*. The other versions of AbGRI3 found in the remaining SGH isolates are all smaller than AbGRI3-2i. They have clearly evolved from AbGRI3-2i, by various deletions of sequence adjacent to copies of IS*26*, as version 1i, 3i and 5i all contain the adjacent chromosomal inversion. Whether AbGRI3-4 is also derived from AbGRI3-2i cannot be discerned, as the inverted chromosomal segment is not present in AbGRI3-4 as its characteristic deletion extends past this region of the chromosome. This deletion has been detected in isolates from Singapore, India (PKAB07) and Sweden (A072) suggesting that the sub-lineage containing AbGRI3-4 has dispersed quite successfully.

Tracking the movement of ESKAPE organisms within and between hospitals or countries is key to effective infection control and the simple set of PCRs we devised to map AbGRI3 variants should assist in distinguishing *A. baumannii* carrying the *armA* gene.

### Conclusions

Five different forms of AbGRI3 were identified in this study. As each variant of AbGRI3 defines a sub-lineage of the GC2 clone, the characterization of the AbGRI3 forms can assist detailed tracking of GC2 isolates locally and globally.

## Supplementary Material

Supplementary Data
