# Supplementary material for: Variants of AbGRI3 carrying the armA gene in extensively antibiotic-resistant Acinetobacter baumannii from Singapore
Source: J Antimicrob Chemother. 2017 Jan 10;72(4):1031–9. doi: 10.1093/jac/dkw542 (PMC5400096; doi:10.1093/jac/dkw542)
Supplement: Supplementary Data [file dkw542_Supp.docx]

**Supplementary data**

**Table S1.** Isolates sequenced for this study

| Strain | Source | Year | Mean  (read depth) | No. contigs | Assembly size  (bp) | N50  (bp) | Accession No.  (reads) | Accession No.  (assembly) |
| --- | --- | --- | --- | --- | --- | --- | --- | --- |
| SGH0701 | Blood | 2007 | 80.7 | 64 | 4,061,936 | 220,457 | ERR246908 | FPGM01000000 |
| SGH0908 | Blood | 2009 | 88.2 | 90 | 3,969,270 | 122,571 | ERR246957 | FPGU01000000 |
| SGH1111 | Sputum | 2011 | 87.1 | 66 | 4,001,907 | 220,529 | ERR246919 | FPGR01000000 |
| SGH1112 | Sputum | 2011 | 89.0 | 66 | 4,001,565 | 291,078 | ERR246911 | FPGO01000000 |
